# Supplementary material for: Examining the Ugandan health system’s readiness to deliver rheumatic heart disease-related services
Source: PLoS Negl Trop Dis. 2021 Feb 16;15(2):e0009164. doi: 10.1371/journal.pntd.0009164 (PMC7909659; doi:10.1371/journal.pntd.0009164)
Supplement: S1 Text — List of legends for S1 Text. S1 Text Table A: Organization of Uganda’s health care system. S1 Text Table B: Indicators used to measure service readiness for three types of RHD interventions. S1 Text Table C: RHD service readiness by district and management authority. S1 Text Table D: Proportion of health facilities reporting availability of all RHD service readiness indicators. S1 Text Fig A: Districts included in the study. S1 Text Fig B: Availability of specific RHD primary prevention indicators, by district. Note: Green bars indicate average scores (out of total possible scores). S1 Text Fig C: Availability of specific RHD management indicators by district. Note: Green bars indicate average scores (out of total possible scores). S1 Text Fig D: Correlation of RHD service readiness score (overall) with general service readiness score obtained from the SARA instrument. Note: General health facility service readiness scores were calculated using instructions published in: WHO (2010). Monitoring the building blocks of health systems: a handbook of indicators and their measurement strategies. Geneva: World Health Organization. S1 Text Fig E: Barriers and enablers to RHD care identified during health work interviews. S1 Text Fig F: Summary matrix of key themes from health worker interviews. (DOCX) [file pntd.0009164.s001.docx]

**S1 APPENDIX**

**A. DATA COLLECTION INSTRUMENTS**

**1. RHEUMATIC HEART DISEASE FACILITY SURVEY TOOL**

**PART 1: SERVICES**

**Primary prevention of rheumatic fever**

**Does this facility offer diagnosis and management services for upper respiratory tract infection (including pharyngitis and tonsillitis)?**

Yes

No

Don’t know

Unable to obtain

**Which types of providers diagnose and treat upper respiratory tract infections?**

Generalist (non-specialist) medical doctors

Specialist medical doctors

Non-physician clinicians/ paramedical professionals

Nursing professionals

Midwifery professionals

Pharmacists

Laboratory technicians (medical and pathology)

Community health workers

Unable to obtain

**Do you have national guidelines for pharyngitis management available in this facility today? (If available ask to see the document)**

Yes, observed

Yes, reported not seen

No

Unable to obtain

**How do providers diagnose likely bacterial pharyngitis?**

|  | Always | Sometimes | Rarely | Never |
| --- | --- | --- | --- | --- |
| Signs and symptoms |  |  |  |  |
| Clinical decision rule( Ask to see a copy and confirm that it is not simply signs and symptoms |  |  |  |  |
| Rapid Strep test |  |  |  |  |
| Throat culture |  |  |  |  |
|  |  |  |  |  |

**Do providers in this facility prescribe antibiotics?**

Yes

No

Don’t know

Unable to obtain

**Do providers in this facility prescribe benzathine benzylpenicillin for pharyngitis?**

Yes

No

Don’t know

Unable to obtain

**What other antibiotics are prescribed for pharyngitis?**

**Which antibiotic would you say was the most commonly prescribed in the past one month?**

**Does this facility dispense antibiotics for pharyngitis?**

Yes

No

Don’t know

Unable to obtain

**Does this facility dispense benzathine benzylpenicillin for pharyngitis?**

Yes

No

Don’t know

Unable to obtain

**Have you or any other provider (s) of services for upper respiratory tract infection received any training in the diagnosis and management of pharyngitis in the past 2 years?**

Yes

No

**Please help me count the number of cases of URTI seen over the past 30 days (Ask to see logbook)**

Number of “URTI” listed as diagnosis

**Please help me count the number of cases of URTI seen over the past 30 days (Ask to see logbook)**

Number with “tonsillitis” listed as diagnosis

**Please help me count the number of cases of URTI seen over the past 30 days (Ask to see logbook)**

Number with “pharyngitis” listed as diagnosis

**Acute Rheumatic Fever (ARF)**

**Does this facility offer diagnosis and management services for acute rheumatic fever?**

Yes

No

Don’t know

Unable to obtain

**Which types of providers diagnose and treat acute rheumatic fever?**

Generalist (non-specialist) medical doctors

Specialist medical doctors

Non-physician clinicians/ paramedical professionals

Nursing professionals

Midwifery professionals

Pharmacists

Laboratory technicians (medical and pathology)

Community health workers

Unable to obtain

**Do you have guidelines for acute rheumatic fever management available in this facility today? (If available ask to see document)**

Yes, observed

Yes, reported not seen

No

Unable to obtain

**Have you or any other provider (s) of services for upper respiratory tract infection received any training in the diagnosis and management of acute rheumatic fever in the past 2 years?**

Yes

No

**Please help me count the number of cases of acute rheumatic fever seen over the past 30 days (Ask to see logbook)**

Number of “ARF” listed as diagnosis

**Secondary prevention**

**Does this facility offer secondary prophylaxis services for prevention of rheumatic fever and rheumatic heart disease?**

Yes

No

Don’t know

Unable to obtain

**Does this facility have national guidelines for secondary prevention available in this facility today? (If available ask to see document)**

Yes, observed

Yes, reported not seen

No

Unable to obtain

**Does your facility keep a list or register of patients receiving secondary prophylaxis? (if available ask to see document)**

Yes, observed

Yes, reported not seen

No

Unable to obtain

**Does this facility regularly audit the register to assess quality of care and adherence?**

Yes

No

Don’t know

Unable to obtain

**Does this facility regularly update the register? (e.g if a patient dies or moves away?)**

Yes

No

Don’t know

Unable to obtain

**Please help me count the number of individuals who have received secondary prophylaxis injections over the past 30 days (ask to see register if exists, or outpatient log book if not).**

**Have you or any other provider(s) of services for acute rheumatic fever received any training in the diagnosis and management of pharyngitis in the past two years?**

Yes

No

**Rheumatic Heart Disease (RHD)**

**Does this facility offer diagnosis and management services for rheumatic heart disease and its complications, such as heart failure, atrial fibrillation, stroke, and infective endocarditis?**

Yes

No

Don’t know

Unable to obtain

**If yes, for how many complications of rheumatic heart disease does this facility offer diagnosis and management services for?**

Some but not all complications

Nearly all complications

Don’t know

Unable to obtain

**Which types of providers diagnose and manage rheumatic heart disease at this facility?**

Generalist (non-specialist) medical doctors

Specialist medical doctors

Non-physician clinicians/ paramedical professionals

Nursing professionals

Midwifery professionals

Pharmacists

Laboratory technicians (medical and pathology)

Community health workers

Unable to obtain

**Do you have national (or international) guidelines for rheumatic heart disease management in the areas of:**

|  | Yes |  | No |  | Don’t know |  | Unable to obtain |
| --- | --- | --- | --- | --- | --- | --- | --- |
| Antenatal care and family planning |  |  |  |  |  |  |  |
| Chronic heart failure including referral for surgical evaluation |  |  |  |  |  |  |  |
| Atrial fibrillation |  |  |  |  |  |  |  |
| Stroke( Note: may have general stroke management guidelines; these are acceptable |  |  |  |  |  |  |  |
| Infective endocarditis including referral for surgical evaluation |  |  |  |  |  |  |  |
|  |  | |  | |  | |  |

**Have you or any other provider(s) of services of rheumatic heart disease received any training in its diagnosis (including cardiac ultrasound) and management during the past 2 years?**

Yes

No

Unable to obtain

**PART 2: DIAGNOSTICS**

**Does this facility offer the following tests onsite or offsite? (Note: Electrocardiography (ECG) and ultrasound already included in the SARA)**

|  | Yes, onsite |  | Yes, offsite |  | Don’t conduct the test |  |  |
| --- | --- | --- | --- | --- | --- | --- | --- |
| Erythrocyte sedimentation rate (ESR) |  |  |  |  |  |  |  |
| C-reactive protein (CRP) |  |  |  |  |  |  |  |
| Anti-Streptolysin O titers ( ASO titers) |  |  |  |  |  |  |  |
| Anti-DNase B(ADB) |  |  |  |  |  |  |  |
| INR |  |  |  |  |  |  |  |
|  |  |  |  |  |  |  |  |

**PART 3: MEDICINES AND COMMODITIES**

**Are any of the following medicines available in the facility today? (Note: BPG, ACEi, ASA, BB, spironolactone, oral furosemide, digoxin already included in the SARA)**

|  | Observed |  | Reported not seen |  | No |  | Unable to obtain |
| --- | --- | --- | --- | --- | --- | --- | --- |
| Injectable furosemide |  |  |  |  |  |  |  |
| Digoxin |  |  |  |  |  |  |  |
| Warfarin |  |  |  |  |  |  |  |
| Heparin by infusion |  |  |  |  |  |  |  |
| Low molecular weight heparin(e.g enoxaparin) |  |  |  |  |  |  |  |

BPG-Benzathine benzylpenicillin G, ASA- Acetyl salicylic acid, ACEi- Angiotensin enzyme inhibitor, BB-Beta blockers.

**2. DISCUSSION GUIDE FOR INTERVIEWS WITH HEALTH WORKERS PROVIDING RHD CARE**

**Objective: Identify local barriers and enablers to RHD prevention and care**

Selection criteria: Key informants (clinical providers) at the regional referral hospital who provide care for patients with RHD, recruited to reflect range of specialization (i.e., pharmacists, nurses, mid-level providers, physicians)

Turn on recorder.

Provide a copy of the informed consent form to the participant. Go through the whole form with them and give them an opportunity to ask questions. If they cannot provide written consent for some reason, tape-record their verbal consent.

Explain the ground rules:

- This is a study to determine how to provide better care for RHD
- There are no right or wrong answers
- Please be frank and share your opinion; the data we gather are confidential
- We will not link your identity to any of your comments in any reports we produce

Q1. Tell me about yourself

- Age,
- Qualification and where training (eg, nursing/medical school) done
- How long qualified, how long working at ### regional referral hospital
- Demographics of patients in your practice and common illnesses treated (by age group)

Q2. Tell me a bit about your training on RHD

- Received any specific training during school?
- Received any training since graduation? If yes, when and where and by whom?
- How many patients with RHD do you see per [day, week, month]? Are these inpatients or outpatients or both?

Q3. Now I’m interested in what you’ve been taught…

- What’s your understanding of cause(s) of RHD?
- Aware of a link between sore throat and RHD?
- What were you taught can be done to prevent RHD? Treat RHD?
- Are there any aspects of RHD management in which you think you need more training?
- What’s your understanding about the long-term prognosis of RHD?

Q4. Tell me about your encounters with patients with RHD…

- (Start by asking them to recall the last patient they saw with RHD and briefly summarize the visit)
- Average age of your RHD patients, working/not working (or not in school)
- Are they generally aware of link between sore throat, ARF, and RHD?
- What sorts of symptoms do they describe?
- Generally good about follow-up and adherence? Or not?
- What sort of barriers do they commonly state to getting the care they need? (**note: will ask about barriers you face, later)
- What sorts of barriers do you perceive they face in getting the care they need?

Q5. Local health system barriers (which of these pieces is not working for you, and why?)

- Administration and leadership in district
- Funding for healthcare in general and RHD in particular (if they are aware of this information)
- Healthcare workers (number and qualification, waiting times, quality of care)
- Medications (particularly BPG, anticoagulation, and heart failure drugs) and diagnostics (particularly echocardiography)
- Health information/medical record systems (integration between facilities; inpatient and outpatient; registers of ARF or RHD patients)
- Guidelines and protocols for RHD care (including referral pathways)

Q6. Local health system enablers (which of these pieces is working for you, and why?)

>> go through categories in Q5 again, looking for enabling factors

Q7. Perception of patient outcomes

- Generally, do you think they get the care they need or not (esp. surgery)? Why?
- Any patient safety and quality-of-care concerns? Preventable deaths in hospital?
- Do you think patients are dying in the community w/o presenting to hospital for care?
- What in your opinion are the 1 or 2 most important things MOH could do to improve outcomes?

Thank participant for their time. Turn off recorder.

Provide opportunity for them to give feedback and ask questions.

Provide them with cash.

Fill out field notes (see below) once they’ve left.

**Field Notes^[[1]](#footnote-1)^**

Setting of interview (geographic location/address and type of building/place):

Any other individuals present during interview? Potential impact they had on discussion?

Appearance and demeanor of participant:

Notable behaviors or nonverbal cues (i.e., not captured in recording/transcript):

Any changes or adaptations of discussion topics, probes, etc. for this particular interview?

Reflections:

- Did the interview feel easy or difficult? Unnecessarily brief or lengthy?
- How did I perform during the interview? Was I successful in eliciting rich, in-depth responses?
- Do I note any potential biases or feelings of mine that came through during the interview and affected the conversation?

Key words and concepts from interview:

| **Level** |  | **Patient, household, and community** | **Provider and health system** |
| --- | --- | --- | --- |
| **Individual** | Barriers |  |  |
|  | Enablers |  |  |
| **Interpersonal** | Barriers |  |  |
|  | Enablers |  |  |
| **Community** | Barriers |  |  |
|  | Enablers |  |  |
| **Organizational** | Barriers |  |  |
|  | Enablers |  |  |
| **Policy & enabling environment** | Barriers |  |  |
|  | Enablers |  |  |

**B. SUPPLEMENTARY TABLES**

**S1 Text Table A.** Organization of Uganda’s healthcare system.

| **Level of health facility** | **Population**  **served** | **Services offered** |
| --- | --- | --- |
| VHT | Village:1,000 | Community based preventive and promotive services |
| HC II | Parish: 5,000 | Preventive, promotive, outpatient curative services |
| HC III | Sub county: 20,000 | Preventive, promotive, outpatient curative services, maternity, inpatient, laboratory |
|  |  |  |
| HC IV | County: 100,000 | In addition to services offered at HCIIIs, emergency surgery and blood transfusion |
| District Hospital | District: 500,000 | In addition to services at HC IVs, in-service training, consultation & research for community-based health care programs |
|  |  |  |
| Regional Referral Hospital | Region: 2.0 million | In addition to services offered at the district hospital, specialist services e.g., psychiatry, ear, nose and throat, ophthalmology, dentistry, intensive care, radiology, pathology, specialised surgical care and medical services |
|  |  |  |
| National Referral Hospital | Country: 39 million | Comprehensive specialist services, teaching and research |

Notes: HC-Health center, VHT- Village health team.

**S1 Text Table B.** Indicators used to measure service readiness for three types of RHD interventions.

| **No** | **Category** | **Primary prevention indicators** | **HCII** | **HCIII** | **HCIV** | **Hospitals** |
| --- | --- | --- | --- | --- | --- | --- |
| 1 | Guideline | National guidelines for pharyngitis management | Yes | Yes | Yes | Yes |
| 2 | Training | Training of staff on pharyngitis management | Yes | Yes | Yes | Yes |
| 3 | Medicine | Observed stock of oral amoxicillin | Yes | Yes | Yes | Yes |
| 4 | Medicine | Observed stock of injectable benzathine penicillin G | Yes | Yes | Yes | Yes |
| 5 | Medicine | Observed stock of oral azithromycin | Yes | Yes | Yes | Yes |
|  | **Category** | **Secondary prevention indicators** | **HCII** | **HCIII** | **HCIV** | **Hospitals** |
| 6 | Guideline | National guidelines for secondary prevention of rheumatic fever/RHD | NA | Yes | Yes | Yes |
| 4* | Medicine | Observed stock of benzathine penicillin G | NA | Yes | Yes | Yes |
| 5* | Medicine | Observed stock of azithromycin | NA | Yes | Yes | Yes |
| 7 | Diagnostic | Availability of functional ultrasound machine | NA | NA | Yes | Yes |
| 8 | Diagnostic | Availability of functional ECG machine | NA | NA | Yes | Yes |
| 9 ** | Diagnostic | Availability of CRP test | NA | NA | NA | Yes |
| 10 | Diagnostic | Availability of ESR test | NA | NA | NA | Yes |
| 11** | Diagnostic | Availability of ASOT test | NA | NA | NA | Yes |
|  | **Category** | **RHD medical management indicators** | **HCII** | **HCIII** | **HCIV** | **Hospitals** |
| 12 | Guideline | National guidelines for RHD management | NA | NA | Yes | Yes |
| 13 | Training | Training of staff on RHD management | NA | NA | Yes | Yes |
| 14 | Training | Training in RHD diagnosis (including ultrasound) and management | NA | NA | Yes | Yes |
| 15 | Medicine | Observed stock of oral furosemide | NA | NA | Yes | yes |
| 16 | Medicine | Observed stock of parenteral furosemide | NA | NA | Yes | yes |
| 17 | Medicine | Observed stock of oral spironolactone | NA | NA | Yes | yes |
| 18 | Medicine | Observed stock of oral digoxin | NA | NA | Yes | Yes |
| 19 | Medicine | Observed stock of any oral ACE inhibitor | NA | NA | Yes | Yes |
| 20 | Medicine | Observed stock of any oral beta blocker | NA | NA | Yes | Yes |
| 21 | Medicine | Observed stock of warfarin | NA | NA | NA | Yes |
| 22 | Diagnostic | Availability of point-of-care INR test | NA | NA | NA | Yes |
| 7* | Diagnostic | Availability of functional ultrasound machine | NA | NA | Yes | Yes |
| 8* | Diagnostic | Availability of functional ECG machine | NA | NA | Yes | Yes |

* Duplicate items that are not double counted in the total score calculation

** A few (private) health centers IV had these laboratory tests available, but these tests were not considered essential for this level of care, so data on the availability of these laboratory tests in these facilities were not included in the calculation of facility readiness scores.

**S1 Text Table C.** RHD service readiness by district and managing authority.

| **District** | **Managing authority** | **Primary prevention** | **Secondary prevention** | **RHD medical management** | **Overall** |
| --- | --- | --- | --- | --- | --- |
|  |  | **%** | **%** | **%** | **%** |
| Lira | Public | 34 | 35 | 28 | 32 |
|  | Other | 54 | 58 | 46 | 52 |
| Wakiso | Public | 49 | 40 | 32 | 41 |
|  | Other | 76 | 59 | 39 | 62 |
| Mbarara | Public | 48 | 38 | 34 | 43 |
|  | Other | 65 | 57 | 82 | 60 |
| Tororo | Public | 25 | 33 | 12 | 21 |
|  | Other | 50 | 52 | 44 | 46 |

Notes: Percentage values are calculated as the average value, across facilities within each stratum, of the ratio of observed facility readiness score to total possible score for each of the three service areas for each facility type. Consistent with the main analysis, if a service area was deemed inappropriate to deliver at a particular type of facility (e.g., RHD medical management at health centers II), then that type of facility was excluded from the calculations. “Other” includes private-for-profit and private not-for-profit facilities.

**S1 Text Table D.** Proportion of health facilities reporting availability of all RHD service readiness indicators.

| **Location** | **Primary prevention** | **Secondary prevention** | **RHD medical management** |
| --- | --- | --- | --- |
|  | **N (%)** | **N (%)** | **N (%)** |
| Lira district | 0 (0.0) | 2 (5.7) | 0 (0.0) |
| Wakiso district | 3 (4.1) | 1 (3.6) | 1 (9.1) |
| Mbarara district | 0 (0.0) | 1 (2.9) | 0 (0.0) |
| Tororo district | 1 (0.6) | 0 (0.0) | 0 (0.0) |
| All four districts | 4 (1.0) | 4 (2.0) | 1 (2.0) |

Notes: “denominators” for proportions include only facilities that “should” be able to provide these three types of interventions. For example, all facilities should be able to provide primary prevention, but only facilities at health center IV or higher level should be able to provide RHD medical management. Proportions can be understood as proxies for “coverage” of RHD interventions in Uganda.

**C. SUPPLEMENTARY FIGURES**

**S1 Text Fig A.** Districts included in this study.

Map created from Wikimedia commons, OpenStreetMap contributors, Jarry1250, NordNordWest/Wikipedia, License: CC-BY SA-3.0


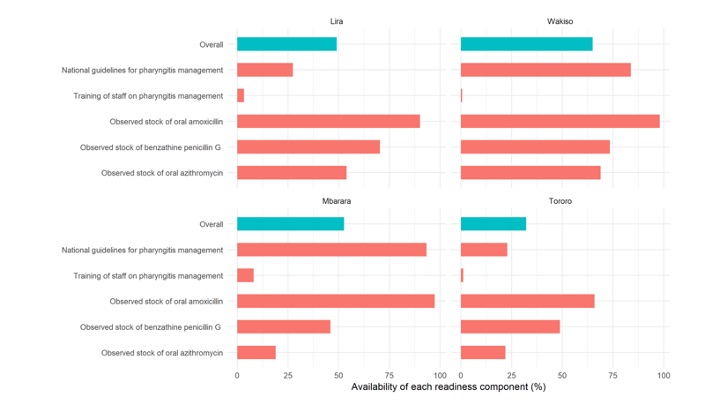


**S1 Text Fig B.** Availability of specific RHD primary prevention indicators, by district. Note: Green bars indicate average scores (out of total possible scores).


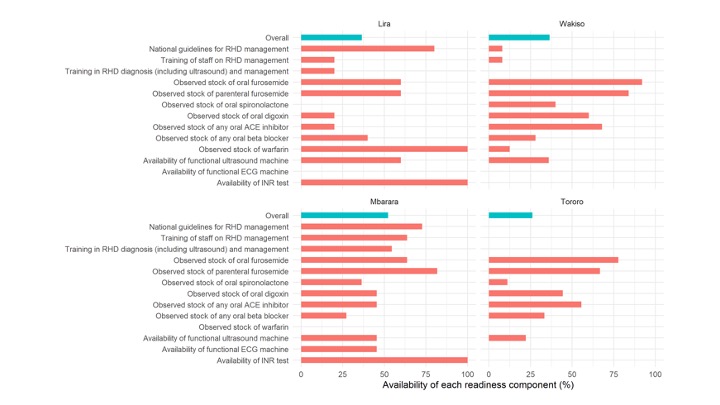


**S1 Text Fig C.** Availability of specific RHD medical management indicators, by district. Note: Green bars indicate average scores (out of total possible scores).


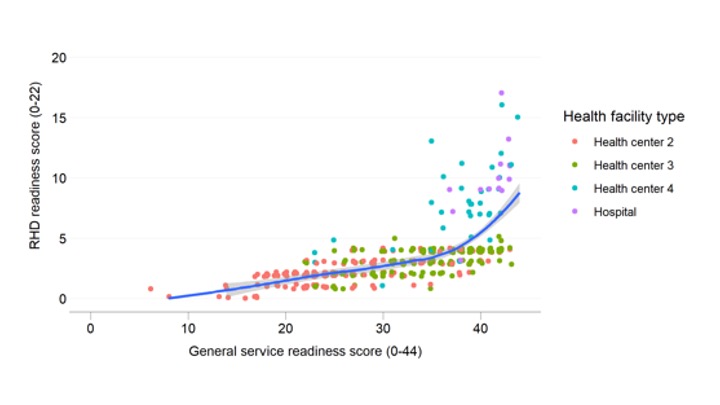


**S1 Text Fig D.** Correlation of RHD service readiness score (overall) with general facility service readiness score obtained from the SARA instrument. Note: General health facility service readiness scores were calculated using instructions published in: WHO (2010). *Monitoring the building blocks of health systems: a handbook of indicators and their measurement strategies.* Geneva: World Health Organization.


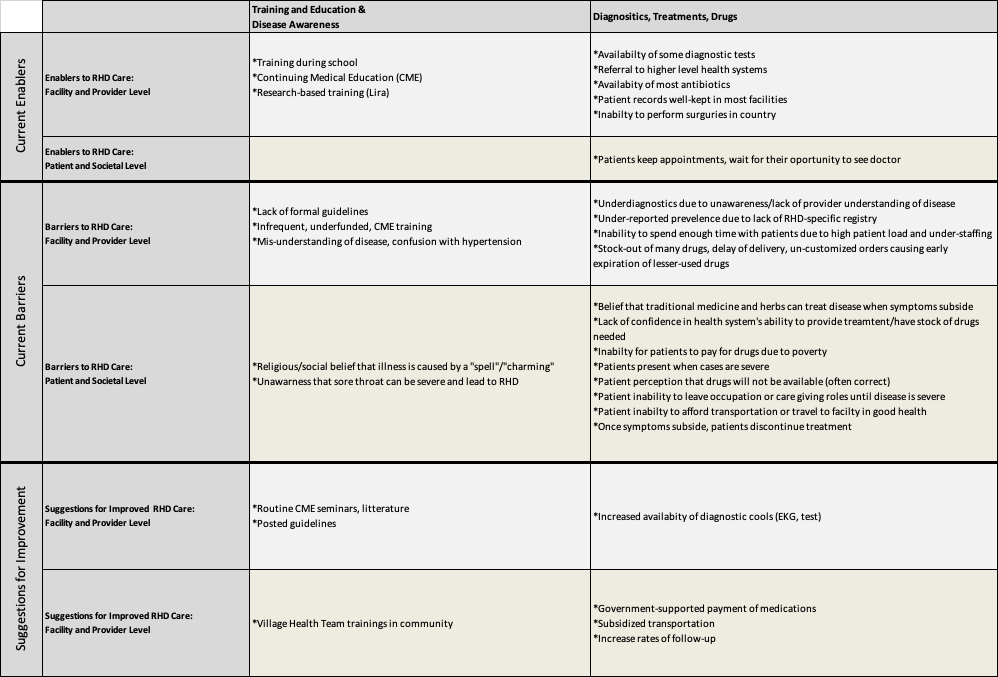


**S1 Text Fig E.** Barriers and enablers to RHD-related care identified during health worker interviews.


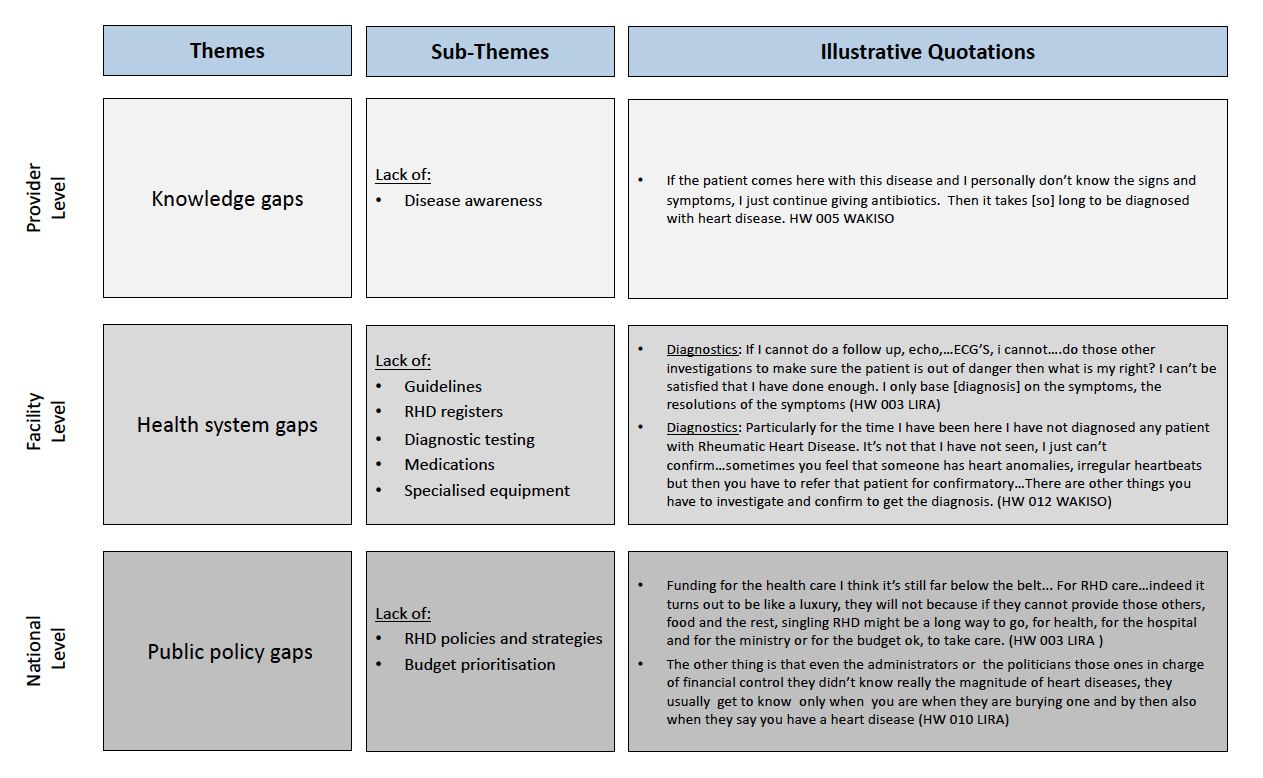


**S1 Text Fig F.** Summary matrix of key themes from health worker interviews.

1. Adapted from Phillipi and Lauderdale, 2018 [↑](#footnote-ref-1)
