# Supplementary material for: Examining the Ugandan health system’s readiness to deliver rheumatic heart disease-related services
Source: PLoS Negl Trop Dis. 2021 Feb 16;15(2):e0009164. doi: 10.1371/journal.pntd.0009164 (PMC7909659; doi:10.1371/journal.pntd.0009164)
Supplement: Attachment — Submitted filename: PLoS NTDs response to reviewers.docx [file pntd.0009164.s002.docx]

David A. Watkins, MD, MPH

Departments of Medicine and Global Health

University of Washington School of Medicine

325 9^th^ Ave Box 359931 | Seattle, WA 98104

December 1^st^, 2020

To the Editors of PLoS Neglected Tropical Diseases,

We received your decision letter regarding our original research article,

“**Examining the Uganda Health System’s Readiness to Deliver Rheumatic Heart Disease related services**”

We have attached the revised manuscript into which we incorporated a number of changes based on the very insightful suggestions made by Reviewer #1. Detailed responses to each of the Reviewer’s points are provided below.

As suggested by the editors, we have also addressed the issue of access to care and the relationship between rheumatic heart disease and the universal health coverage agenda in Uganda.

Thank you very much for giving us the opportunity to revise and resubmit our manuscript. We look forward to your decision soon.

Sincerely,

David Watkins

Assistant Professor of Medicine and Global Health

University of Washington

[davidaw@uw.edu](mailto:davidaw@uw.edu)

**Responses to comments from the Editors**

I would ask you to careful consider the suggestions of the reviewer, and also to discuss a point that is absent from your text. Availability and access to health care resources are very different things and most of your discussion refers to the availability of specific resources.

To the best my knowledge, in Uganda, even where high complexity procedures are available (for example, surgery or valvoplasty), patients may do not have access to them because they would need to pay for the service. The WHA resolution is very clear that access is the major issue here, and I strongly suggest the authors to discuss a little more about this question.

Universal health coverage seems to be essential to guarantee access to health care in all levels.  There was an UN General Assembly high-level meeting on universal health coverage in September 2019 and the resolution calls on Member States to accelerate progress towards universal health coverage with a focus on poor, vulnerable and marginalized individuals and groups.

Thank you for raising this important point. We completely agree that improving access to RHD-related care in Uganda is about more than just improving the availability of resources. The broader Ugandan RHD research program sponsored by the American Heart Association includes a number of projects that are seeking characterize access barriers (including economic barriers such as out-of-pocket costs) and pilot alternative service delivery approaches that achieve better access as well as higher quality of care. While the findings of these projects are outside the scope of the submitted manuscript (and are ongoing), we agree that the discussion section needs to better contextualize our findings and their limitations. On p. 20 we have added the following paragraph:

*Our study should also be placed in the broader context of RHD-related health services research in Uganda. We focused on measuring the availability of inputs and resources needed for RHD-related healthcare. Other gaps in healthcare access and quality, which we did not attempt to ascertain in this study, would present additional challenges to achieving universal health care coverage in the context of RHD. Remediation of access barriers will not only require “supply-side” solutions (e.g., improving availability of healthcare inputs), which we emphasize here, but also “demand-side” solutions that address deterrents to health-seeking behavior. For example, ongoing research by our group has begun to shed light on the significant transportation barriers and catastrophic out-of-pocket costs faced by Ugandans with RHD.[25] Additionally, solving all access barriers does not ensure that RHD outcomes will be improved: Low quality of care potentially wastes scarce resources and undermines trust in health care systems. Patient uptake of penicillin prophylaxis and appropriate use of medications like warfarin have been documented to be low, even among those receiving care at tertiary academic hospitals.[26] The findings of this study are a necessary starting point for developing national RHD strategies and policies, but achieving universal health coverage in the area of RHD will require additional assessments and health system reforms and policies that are more comprehensive.*

**Detailed responses to comments from Reviewer #1**

Abstract

    1.  Methods. The authors report employing a “standardized instrument” for the health facility survey. Consider stating from where this instrument came or was adapted.

We used the WHO SARA tool. We have specified this in the revised abstract.

    2.  The sentence starting with “Health worker interviews perceived” should be rephrased or “perceived” should be replaced by another work such as “revealed” or “demonstrated.” It is the health workers who perceive, not the interviews themselves.

Thanks for the suggestion. The word perceived has been replaced with revealed.

    3.  In the Conclusion section, is “education” the best word or is “specialized training” more representative of the message?

We have changed this to “training.”

Author Summary

    1.  “how to address this problem” is slightly misleading – the study is about assessment of existing infrastructure/exposing health system gaps. While areas of need are identified, the study does not specifically posit or test strategies or frameworks for implementation.

We have changed this to “assessment of health systems gaps in delivery of RHD related care.”

    2.  The sentence beginning with “Our approach used” would benefit from rephrasing using less colloquial language.

We have changed the statement “Our approach used” to “We used.”

    3.  “have everything they need” could be rephrased to read “fully equipped”.

We have rephrased this to “fully equipped.”

    Introduction

    1.  Page 1 lines 6-9. The authors enumerate 2 strategies to prevent RHD. However, primordial prevention is not mentioned. Primordial prevention of RHD through socioeconomic advancement is one of the primary drivers of the previously mentioned eradication of RHD in wealthy nations. Consider either mentioning primordial prevention, or emphasizing that the 2 strategies noted are those most amenable by the health system.

A statement on primordial prevention has been added.

    2.  Page 1 line 10-11: can remove this sentence or move to later in paper to improve flow in introduction

Several edits have been made to the introduction to improve its flow.

    Methods

    1.  Page 2-3 line 51-54. The authors mention districts were chosen because of specific features including representative of the general population and because existing studies were being performed. Does the active research programs influence health system/staff preparedness for RHD care?

We acknowledge that it might, and we raised this in the discussion (p. 16). However, because the research programs were being conducted primarily as epidemiological investigations, without an explicit partnership with the Ministry of Health to scale up access to RHD-related care, we are very confident that any influence the research had would be limited to provider awareness and would not extend, for instance, to availability of medicines and diagnostics at health facilities. We have added this clarification (also on p. 16).

    2.  Page 3, line 70. How many health facilities were invited from the census? What is the response rate?

This information was included in our results section but has been moved to the methods section and expanded (on pp. 3-4) for clarity.

    3.  Page 3, line 76. SARA acronym is the Service Availability and Readiness Assessment. The term “service” is omitted. Please correct.

This has been corrected.

    4.  Page 4, line 85. Please emphasize the data collection included both verbal/written response of respondents, as well as direct observations at the health facility. This is not clear as written.

This has been emphasized as suggested.

    5.  Page 4, line 91. How did the study team ensure data entry was of high quality and free of entry errors?

Data was checked for correctness and errors by two members of the research team (EN, JA) on a daily basis. This has been indicated in the revised manuscript (p. 5).

    6.  Page 5 line 107-108: remove parentheses

Parentheses have been removed.

    7.  Page 5, line 114. Was participation by providers voluntary? If so, this should be indicated. Currently there is a statement that facility representatives agreed to participate but not indicated that recruitment was free of coercion.

We have indicated in the revised manuscript that participation of respondents was voluntary and free of coercion.

    8.  Page 5 line 115. Why did the authors decide to interview providers only at the highest level health facility in each of the 4 districts? Why not sample from a range of health facility types, including lower levels where most primary prevention activities would be conducted?

Health care providers at the highest level of health facility are more likely to interact with RHD patients hence are more aware of the challenges/barriers faced in management of these patients. Prior experience of the research team working in these districts suggested that most primary healthcare staff were unaware of rheumatic fever and RHD and would therefore not yield informative data about health system barriers. This justification was included in the revised methods section (p. 6).

    9.  Page 5 line 119: “no connection” – does this refer to conflict of interest?

Yes, this refers to conflict of interest, and this has been clarified.

    10. Page 5 line 123: Was English the primary language of interviewed health workers? It would be good to include a statement of understanding of health workers of interview questions and whether any translation to the local language was required.

Although English was not the primary language of the health workers interviewed, translation to local languages was not necessary since English is an official language used in various institutions in Uganda. A statement to this effect has been added to the methods section (p. 6).

    11. Page 5 line 124. The authors describe this study as a sequent exploratory mixed methods study. Generally, the quantitative data will inform the qualitative data collection. In what way did the quantitative inform the development of the semi-structured interview guide? Or was this more of a triangulation design?

Thank you for requesting clarity on this point. Our terminology regarding study design was somewhat imprecise. We employed *concurrent* data collection, with the facility survey being the primary component (i.e., QUAN + qual), but *dependent* data analysis, with the findings from the survey analysis informing the analysis of interview transcripts. Concurrent data collection was employed for logistical and budgetary reasons. We originally used the phrase “explanatory sequential” on the basis of the *dependence of the data analysis* but acknowledge this could be confusing to some readers, who might assume data were collected sequentially.

As the reviewer may know, there is some debate in the mixed methods literature about the best study design terminology. We think of triangulation as more of a rationale or purpose for pursing a mixed-methods vs single-method design, rather than a design type itself. Our rationale was not triangulation (since we believed the survey results would stand on their own); rather, we believed that qualitative data would enhance and provide further depth to the survey data. (For example, the low availability of diagnostics and cardiac medicines in the survey was explicitly linked – by one of the interviewees – to district and national budgetary decisions and lack of acknowledgement of RHD as a priority.)

To address the reviewer’s concern, we have adopted the typology proposed by Morse and Niehaus while explaining the rationale for the mixed design (p. 2).

    12. Page 6 line 134. The authors state they used a pre-written deductive codebook for the qualitative analysis. What prior conceptions or frameworks formed the basis for the pre-written codebook? The authors later reference the socioecological framework in the results.

The socioecological model was used to inform the pre-written code book. Preliminary findings from the survey analysis also informed the coding process and aggregation of codes into themes. This point has been clarified in the methods section (p. 7).

    13. Page 6. Ethics. Was there ethics approval at Children’s National Hospital – where the REDCap data were stored?

We did not seek IRB approval at Children’s National Hospital. It is not mandatory to specifically have approval at Children’s National Hospital for one to use their Redcap database, so long as documentation is provided of IRB approval elsewhere. The reason that this particular Redcap was used was purely pragmatic: Children’s National Hospital was the prime for the main grant from the AHA (PI: Dr. Sable), with Drs. Watkins and Beaton being PIs/sub-awardees conducting population and clinical research projects (respectively) that employed the same research staff at the Uganda Heart Institute. It was much easier for the staff at Uganda Heart Institute to use one institution’s Redcap for all their data collection rather than split across three institutions. We did not think that this explanation was necessary to add into the manuscript, but if the Reviewer or Editors wish for us to provide this information in the text to alleviate any ethics concerns, we are happy to do so.

    14. Do the authors have a definition of rural in terms of population? It may be helpful for the reader for this to be specified either in the text or in the caption of Table 1.

Thanks for this suggestion. We defined rural in terms of health facility location. In this study, “rural” refers to health facilities located outside urban centers in each district. The footnote to Table 1 has been modified to clarify this point.

    Results

    1.  Page 9 table 2. Point-of-care INR tests not available at hospitals. Are there other types of INR test platforms?

We did collect data on standard laboratory-based INR testing (2 of 14 hospitals offered this), but we chose to emphasize point-of-care testing because this technology would have greater potential to decentralize to lower-level health facilities without laboratories. However, in hindsight we acknowledge that both types of INR testing should have been mentioned. The text (p. 12) and footnote to table 2 (p. 10) have been modified accordingly.

    2.  Page 10 line 197-198: please specify the name of the test (ASOT?)

This has been specified as suggested.

    3.  Page 11 line 225. The authors report comparing RHD service readiness with general facility service readiness. This is not described in the methods.

Supplementary Fig S4 in the appendix notes that “General health facility service readiness scores were calculated using instructions published in: WHO (2010). Monitoring the building blocks of health systems: a handbook of indicators and their measurement strategies. Geneva: World Health Organization.” We now include this detail in our methods section (p. 5).

    4.  Page 12 line 236. Authors report “higher number of nurses in a health facility was also associated with a significantly higher RHD readiness score.” In table 3, the OR for this relationship is 1.0 with 95% CI 1.0, 1.0; p=.015. Is there a typo in the table, or is does the written statement not reflect the data.

This was an artifact of rounding all estimates to 2 significant digits. The revised table contains estimates rounded to 3 significant digits, which gives a rate ratio of 1.01 with 95% CI 1.00, 1.02.

    Discussion

    1.  Page 15 line 300: “We documented low availability of RHD-related services in four representative districts…” As stated previously, I have reservations describing the 4 districts as “representative”. In fact, the authors state this later in the discussion (line 368-370).

We have removed the word “representative.”

    2.  Page 16 line 316-317: public education is important but seems a bit tangential to the overall message as the study focuses on the health system. Suggest removing or moving to a later section in order to streamline the message in that particular section. The message about readiness is important and adding statements about public education detracts a bit from the overall message

Agree. This has been removed and the sentence re-worked to focus on district-level provider awareness.

    3.  Page 17 line 347: use of the word “pathology” is unclear here – does this refer to tissue pathology, laboratory services or treatment of RHD in general?

The word “pathology” has been replaced with “laboratory services.”

    4.  Page 17 line 350-362: Suggest abbreviating this paragraph to improve flow of the discussion

This has been abbreviated as suggested in the reviewer’s next comment (pp. 18-19).

    5.  Page 17 line 359-361. Authors report that medical management of complicated RHD is not cost-effective in the absence of cardiac surgery. No reference is provided. Please provide a reference. I suspect more appropriate term instead of “cost-effective” would be “prudent”.

To our knowledge, no cost-effectiveness studies have been done on medical management of RHD apart from surgery. We agree that we overstated the point and have re-worded the text accordingly.

    6.  Page 18 line 371-374: parentheses can be removed here

Parentheses have been removed.

    7.  One factor that may or may not be a source of bias in the survey and interviews was pressure by providers based at health centers/hospitals to make facilities seem more equipped, in other words better, than they actually were. Was this a limitation of the study?

Thank you for raising this concern. We do not think that this was a source of bias since the respondents did not have prior knowledge of the specific questions that were to be asked, and before the interview, they were informed that this was not a supervision visit. Most importantly, the research team conducted a visual confirmation of all commodities present or absent at the time the surveys were filled out. The latter detail has been added to the methods section (p. 4).

    8.  Do the authors have a sense of whether the incomplete penicillin stock was due to frequent stock outs, refrigeration issues, or road/transportation issues? This would be interesting to add to the discussion.

Penicillins are one of the most common prescribed antibiotics at the health facilities. Since this was a cross-sectional study, it is possible that at the time of the study there were stock-outs at a number of health facilities. However, this is a conjecture; we did not ask respondents about the reasons for incomplete stock. It will be necessary to conduct an in-depth analysis of supply chain issues at the district, regional, and national levels before planning the scale-up of RHD preventive interventions in Uganda. We now acknowledge this as a study limitation and a direction for future research (p. 17).

    9.  Page 19 line 393. The authors earlier question the utility of pre-service vs. in-service training, but do not mention in-service training here. Continuing training is probably more important than pre-service training in maintaining high quality care.

In-service training has been added to this section.

    Tables

    1.  Table 2: Does NA indicate that the variable did not exist at the particular health facility or that it was not expected to exist? This should be clarified. If the resource was expected to exist but not in stock, this should probably be represented by 0 rather than NA

NA indicates that the variable was not expected to exist at that particular type of health facility. This has been clarified in the table’s footnote.
